# The Unexpected Essentiality of *glnA2* in *Mycobacterium smegmatis* Is Salvaged by Overexpression of the Global Nitrogen Regulator *glnR*, but Not by L-, D- or Iso-Glutamine

**DOI:** 10.3389/fmicb.2018.02143

**Published:** 2018-09-11

**Authors:** Nadya Rakovitsky, Michal Bar Oz, Karin Goldberg, Simon Gibbons, Oren Zimhony, Daniel Barkan

**Affiliations:** ^1^Koret School of Veterinary Medicine, The Robert H. Smith Faculty of Agriculture, Food and Environment, Hebrew University of Jerusalem, Jerusalem, Israel; ^2^Department of Pharmaceutical Engineering, Azrieli College of Engineering, Jerusalem, Israel; ^3^Research Department of Pharmaceutical and Biological Chemistry, UCL School of Pharmacy, London, United Kingdom; ^4^Kaplan Medical Center, Rehovot, Israel; ^5^The Faculty of Medicine, Hebrew University of Jerusalem, Jerusalem, Israel

**Keywords:** mycobacteria, glutamine, glutamine synthetase, nitrogen, metabolism

## Abstract

Nitrogen metabolism plays a central role in the physiology of microorganisms, and Glutamine Synthetase (GS) genes are present in virtually all bacteria. In *M. tuberculosis*, four GS genes are present, but only *glnA1* is essential, whereas *glnA2* was shown to be non-essential for *in-vitro* as well as *in-vivo* growth and pathogenesis, and is postulated to be involved in D-glutamine and iso-glutamine synthesis. Whilst investigating the activity of an antimicrobial compound in *M. smegmatis*, we found a spontaneous temperature-sensitive mutant in *glnA2* (I133F), and used it to investigate the role of *glnA2* in *M. smegmatis*. We deleted the native *glnA2* and replaced it with a mutated allele. This re-created the temperature sensitivity—as after 3–4 seemingly normal division cycles, *glnA2* became essential for growth. This essentiality could not be salvaged by neither L, D- nor iso-glutamine, suggesting an additional role of *glnA2* in *M. smegmatis* over its role in *M. tuberculosis*. We also found that overexpression of the global nitrogen regulator *glnR* enabled bypassing the essentiality of *glnA2*, allowing the creation of a complete deletion mutant. The discrepancy between the importance of *glnA2* in Mtb and *M. smegmatis* stresses the caution in which results in one are extrapolated to the other.

## Introduction

Nitrogen metabolism is important for all bacteria, from harmless soil organisms to pathogens, including various mycobacteria. The synthesis of glutamine is a key metabolic pathway, and in many bacteria, a sole glutamine synthetase (GS, usually called *glnA*) is present, is essential, and its loss leads to glutamine auxotrophy (Merrick and Edwards, 1995; Weisschuh et al., 2000). In *M. tuberculosis*, four GS genes exist, called *glnA1, A2, A3*, and *A4*. Deletion mutants of each of these genes were previously constructed, but only *glnA1* (*Rv2220*) was found to be essential; the deletion mutant was a glutamine auxotroph, and as such, was unable to proliferate in macrophages or mice, and was comparable to BCG as a vaccine candidate in mice (Tullius et al., 2003; Harth et al., 2005; Lee et al., 2006). However, deletion mutants of the other GSs [*glnA2(Rv2222c), glnA3* (*Rv1878*), and *glnA4* (*Rv2860c*)] were fully viable even without glutamine supplementation. Unlike the other *glnA* genes, *glnA2* is sometimes thought to be responsible for synthesis of D-glutamine and possibly iso-glutamine, but still, a deletion mutant could grow in un-supplemented media and replicate in mice (and cause death) with no apparent defect as compared to wt (Tullius et al., 2003; Harth et al., 2005).

Homologues of all four *glnA* genes exist in *Mycobacterium smegmatis*, but their role there has been investigated to a lesser extent. In this study, we started by screening for spontaneous mutants that would be resistant to the chemical substance SG-1, previously described. We isolated several resistant mutants, and sequenced their genomes to try to pinpoint the mutation responsible for the resistance phenotype. We discovered that a point mutation in the *glnA2* gene of *M. smegmatis* (*Msmeg_4294*, homolog of *Rv2222c*) rendered the bacterium temperature-sensitive, and decided to use this finding to explore the role of *glnA2* in *M. smegmatis*.

### Results

#### An I133F mutation in *glnA2* renders the bacterium temperature sensitive

**Figure 1:**
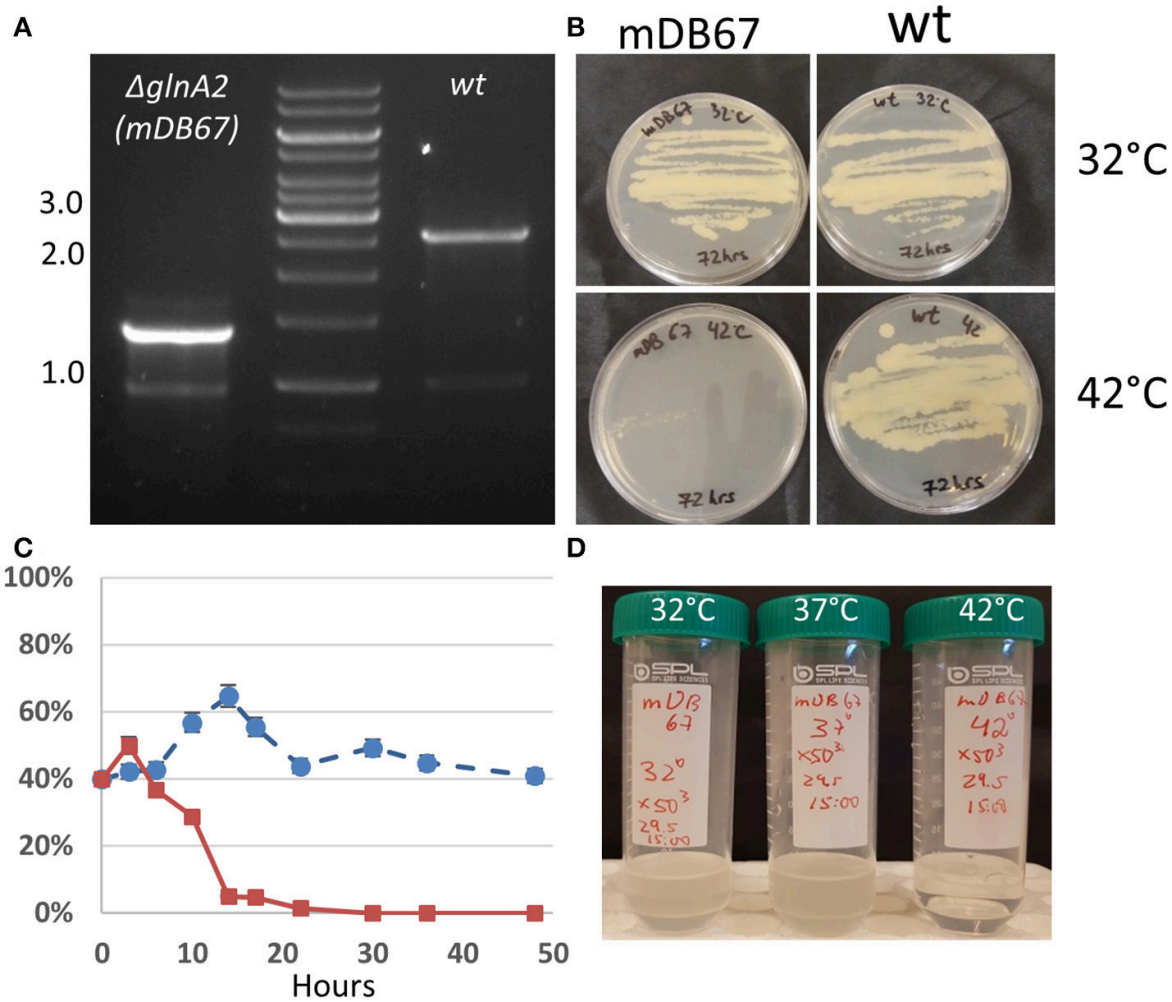
*M. smegmatis glnA2*^I133F^ (mDB67) is temperature sensitive. **(A)** Confirmation of *glnA2* deletion. PCR was done with primers producing a 2.6 kb fragment in wt (right), and 1.3 kb fragment for a complete deletion. The 1.3 kb fragment was also examined by Sanger sequencing. **(B)** 5 × 10^6^ cfu/mL of wt or mDB67 were plated at 32°C or 42°C. mDB67 failed to grow at 42°C. **(C)** A growth-competition experiment of mDB67 vs. wt (mDB149) at 32°C (circles, broken line) and 42°C (squares, solid line). The graph shows the percentage of mDB67 in the mDB67:wt mixed culture. After 3–4 generation times, at 42°C mDB67 rapidly disappears from the mixture. At 32°C, both strains grow equally. **(D)** mDB67 was diluted to 1 cfu/μl in a volume of 30 ml. the culture was split to 3 tubes, and left to grow at 32, 37, or 42°C. the picture was taken after 48 h.

During the study of an antimicrobial compound derived from *Allium stipitatum* that is active against mycobacteria, we isolated a spontaneous *M. smegmatis* mutant that was both resistant to the compound and temperature sensitive in the sense that it grew at 32°C, but not at 42°C. We performed whole genome sequencing on this mutant, and found five point mutations as compared to the parent wt *M. smegmatis* mc^2^-155. One of these point mutations was at the 397th base of the *glnA2* gene, replacing the A with a T, causing an I133F amino acid change. To test if this mutation was involved in the drug resistance phenotype, the temperature sensitivity, or both, we decided to replace the *glnA2* gene in wt mc^2^-155 with the mutated gene. To do this, we cloned a mutated *glnA2* (*glnA2*^*mut*−*I*133*F*^) into a kanamycin-selected, *attp*-integrating vector (pDB221) and electroporated it into wt *M. smegmatis*, creating a merodiploid mutant for *glnA2* (*glnA2*^*wt*^ and *glnA2*^*mut*^). From this merodiploid mutant we deleted the native *glnA2*^*wt*^ using the two-step allelic exchange technique (and plasmid pDB240) (Figure 1A), thus creating a mutant with only a *glnA2*^*mut*^ copy (mDB67, *M.smeg*^*I*133*F*^). This mutant was not resistant to the antimicrobial compound we tested, suggesting that the resistance mechanism was either not related to the mutation, or involved a combination with any of the other identified point mutations. However, the *M.smeg*^I133F^ mutant was temperature sensitive, as it grew normally at 32°C, but not at 42°C, suggesting the function of GlnA2 was essential to normal growth, contrary to the situation in Mtb (Figure 1B).

#### Determination of the death rate of *M.smeg*^I133F^ at 42°C

To further clarify the kinetics of death of mDB67 at 42°C, we decided to conduct a “competition” experiment of mDB67 vs. the *M. smegmatis* wt-like mutant (mDB149, a kanamycin resistant, luminescent mutant). Both mutants were separately grown to log-phase (OD_600_ = 0.2), mixed together in a 1:1 ratio, the mixture diluted X10^−5^, and left to grow in 7H9 media with kanamycin at either 32 or 42°C. Every 3 h at first, and then in longer intervals, a sample was plated, and the ratio of luminescent bacteria (wt, mDB149) to non-luminescent (mDB67) was determined by colony observation. Luminescence was visualized with an IVIS machine. As the generation time of *M. smegmatis* is approximately 3–4 h, it was evident that during the first 2–3 generations mDB67 and mDB149 grew in a similar fashion both at 32 and 42°C (Figure 1C). However, after 10 h, the percentage of mDB67, that remained fairly constant at *circa* 50% at 32°C, was sharply reduced at 42°C to barely detectable levels. At 24 h, the culture at 42°C was completely dominated by mDB149 (with no detectable mDB67), whereas at 32°C, the ratio of 50% remained unchanged for at least 48 h. This growth pattern (normal for 2–3 generations, then a sharp deterioration) suggested either one of two situations: a lack of some vital metabolite or co-factor - at first, the amount of the metabolite was sufficient, but after 2–3 divisions, the consumption and/or dilution of the metabolite was reduced to below critical levels, and there was either growth arrest, or lysis of the cells. Alternatively, a metabolic bottleneck created by an inactive/low activity *glnA2* could prompt the accumulation of an as-yet unidentified metabolite with a toxic effect, reaching lethal levels after approximately 3 generation times.

To more exactly pinpoint the temperature requirements of mDB67, we diluted a 30 ml culture to 1 cfu/μl (confirmed by plating 100 μl on a plate for colony count), split it into three identical tubes of 10 ml each, allowed it to grow for 24 h, and then plated it in dilutions for a cfu count. At 37°C the culture grew to 65 cfu/μl (a generation time of approximately 4 h), at 32°C cfu/μl grew to 20 cfu/μl (generation time ~ 6 h), whereas in the 42°C tube there were no viable bacteria (detection limit was 0.05 cfu/μl). After additional 24 h, the tubes were photographed (Figure 1D).

#### *glnA2* cannot be deleted from *M. smegmatis*

**Figure 2:**
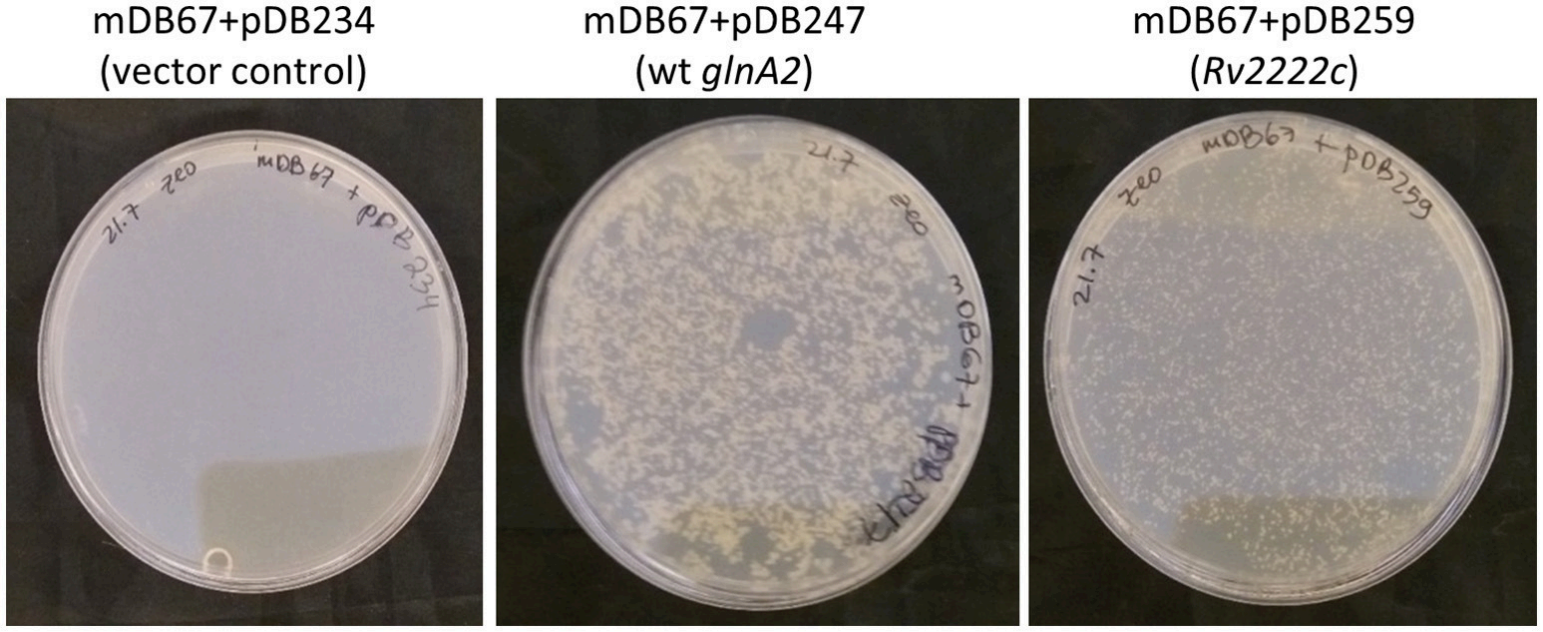
*glnA2* is essential, as it cannot be completely removed from the *M. smegmatis* genome. mDB67 (Δ*glnA2*; attp:kana:*glnA2*^I133F^) was electroporated by an empty control vector (left), wt *glnA2* or *Mtb_glnA2* (*Rv2222c*, right), and plated on 7H10 plates supplemented by oADC, glycerol and zeocin 33 μg/mL.

To further show that *glnA2* is indeed essential in *M. smegmatis*, we attempted to completely remove it, by exchanging the complementing cassette in mDB67 *(M.smeg*^*I*133*F*^) for either one of three zeocin-selected plasmids: pDB234 (empty vector), pDB247 (vector+wt *Msmeg_glnA2*) and pDB259 (vector+*Rv2222c*, the Mtb homolog of *glnA2*). As anticipated, we obtained multiple colonies with the pDB247 plasmid (as this simply reverted the bacteria to the wt genotype). We also obtained multiple colonies with pDB259 (albeit the colonies appeared a day later, and where somewhat smaller). In contrast, no zeocin-resistant colonies could be obtained with the empty vector pDB234 (despite lengthy incubations of up to 14 days), strongly suggesting that the function of GlnA2 is essential (Figure 2). The attempts to electroporate pDB234 (thus obtaining a complete deletion mutant) were repeated several times, but the few colonies that did appear on plates failed to grow in subculture (except for one colony—see analysis below). We also did not obtain colonies with two additional plasmids namely pDB294 and pDB295 [with *Rv1878* (*glnA3*) and *Rv2220* (*glnA1*), respectively], suggesting that these genes could not compensate for the loss of *glnA2* either (data not shown).

These results, together with the lack of growth of mDB67 at 42°C, suggested that in *M. smegmatis glnA2* is an essential gene, whose function can be partially compensated for by the Mtb homolog (*Rv2222c*), but not by other Mtb_*glnA* genes.

#### The essentiality of *glnA2* cannot be salvaged by L-, D-, Iso-glutamine, nor by other supplements

**Figure 3:**
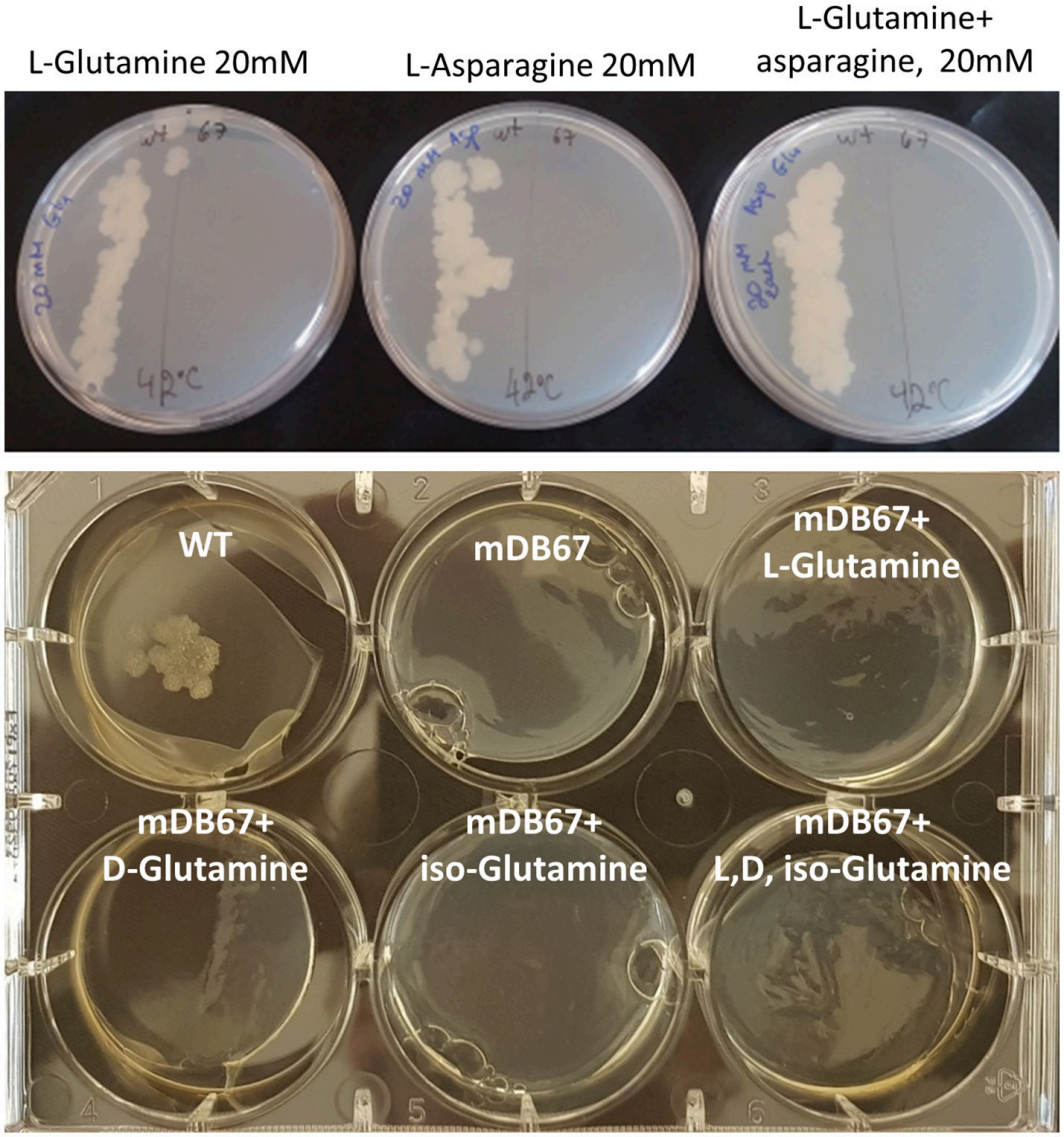
The *glnA2*^I133F^ mutant cannot be salvaged by L, D, iso-Glutamine, nor by all three together. **Top:** wt Msmg plated on the left and mDB67 on the right part of each plate supplemented with the indicated amino acids at the non-permissive temperature 42°C. **Bottom**: WT Msmeg or mDB67 were plated (~50 cfu) at 42°C on 7H10 plates with the indicated amino acid at 20 mM, including with all three (bottom right). At 32°C, mDB67 grew well at all the wells (not shown).

In Mtb, the *glnA2* deletion mutant grew normally and was not a glutamine auxotroph, in contrast to the *glnA1* deletion mutant, which is a glutamine auxotroph. To test if in *M. smegmatis glnA2* deletion causes glutamine auxotrophy, we plated *M.smeg*^*I133F*^ and Mc^2^-155 (wt) at 32 or 42°C, on regular 7H10 plates, or plates supplemented by glutamine, asparagine or both. Neither supplementation salvaged *M.smeg*^*I133F*^ growth at the non-permissive 42°C temperature (Figure 3, Top), suggesting that the growth defect was related to some other, unidentified, function of this gene/enzyme. Since some data have suggested that Mtb_*glnA2* is responsible for the synthesis of D-glutamine or iso-glutamine, and not L-glutamine, we attempted to grow *M.smeg*^*I133F*^ with D-glutamine, but this did not salvage the temperature-sensitivity phenotype either (Figure 3, Bottom).

#### Upregulation of the global nitrogen regulator *glnR* enables complete *glnA2* deletion

**Figure 4:**
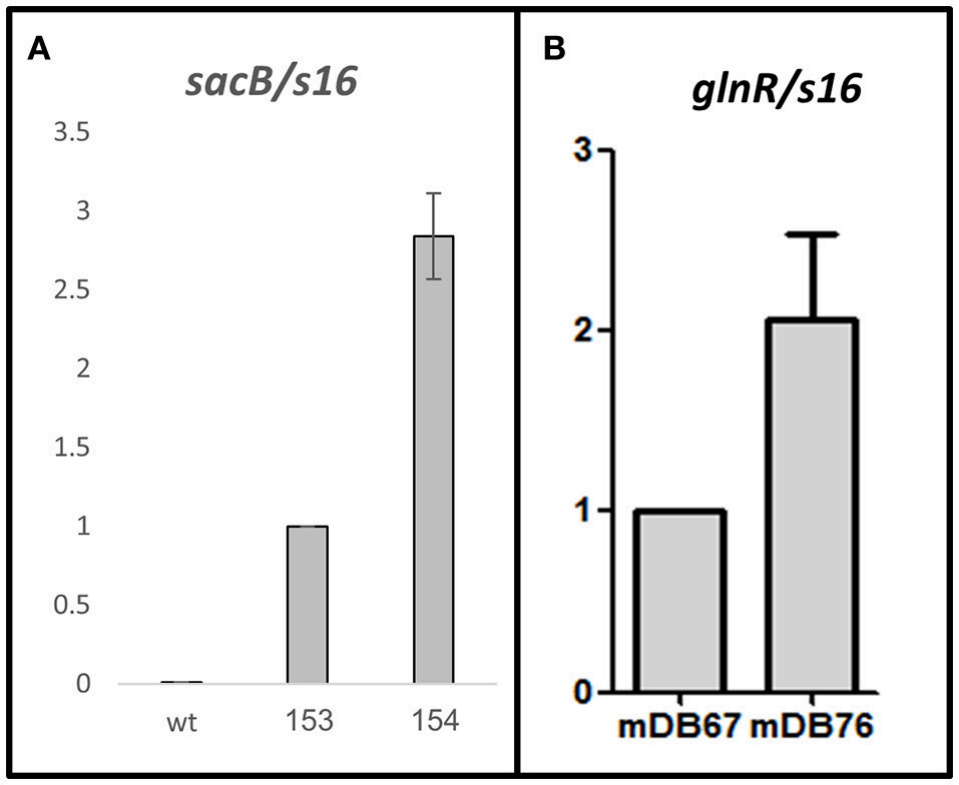
An A=>G mutation in position (-5) at the promoter of *M_smeg*5784 gene (*glnR*) causes up-regulation of the gene. **(A)** *sacB* was used as a reporter gene. No transcript was detected in the wt Msmeg, the transcription level in mDB153 (with *sacB*, single copy, under the wt-*glnR* promoter) was set as 1, and compared to the transcription level in mDB154 (*sacB* under the mutated promoter). **(B)** Transcript levels of *glnR* were measured in mDB67 (set as “1”) and mDB76. For **(A,B)**, mean values of five independent experiments are shown, error bars are SEM.

In one of our repeated attempts to completely delete *glnA2* from *M.smeg*^*I133F*^ by cassette exchange, we obtained a mutant where the cassette exchange was confirmed, and was thus a full *glnA2* deletion mutant. We postulated that the deletion was made possible due to a compensating mutation, and sent the full-deletion mutant (called mDB76) for whole genome sequencing. The only mutation discovered (except for the full absence of *glnA2*) was a point mutation A=>G at position minus 5 (5 bp before the start codon) in *M_smeg 5784*, which is also called *glnR*. This gene was previously shown to be a global regulator-activator of nitrogen metabolism, and involved in the regulation of hundreds of genes (Amon et al., 2008; Malm et al., 2009; Jenkins et al., 2013; Liao et al., 2015). We suspected that the finding of the A=>G point mutation right before the ATG of this particular gene in a *glnA2* full deletion mutant was not a coincidence, and decided to first examine the effect of the mutation on *glnR* promoter activity. We cloned the *sacB* gene under either the native *glnR* promoter (*sacB*^*glnR*−*prom*^) or under the mutated one (*sacB*^*mut*−*glnR*−*prom*^), constructed the respective *M. smegmatis* mutants (mDB153 and mDB154), and examined the transcription levels by RT-PCR. We found that transcription with the mutated promoter was approximately 3-fold higher than that with the wt (Figure 4).

**Figure 5:**
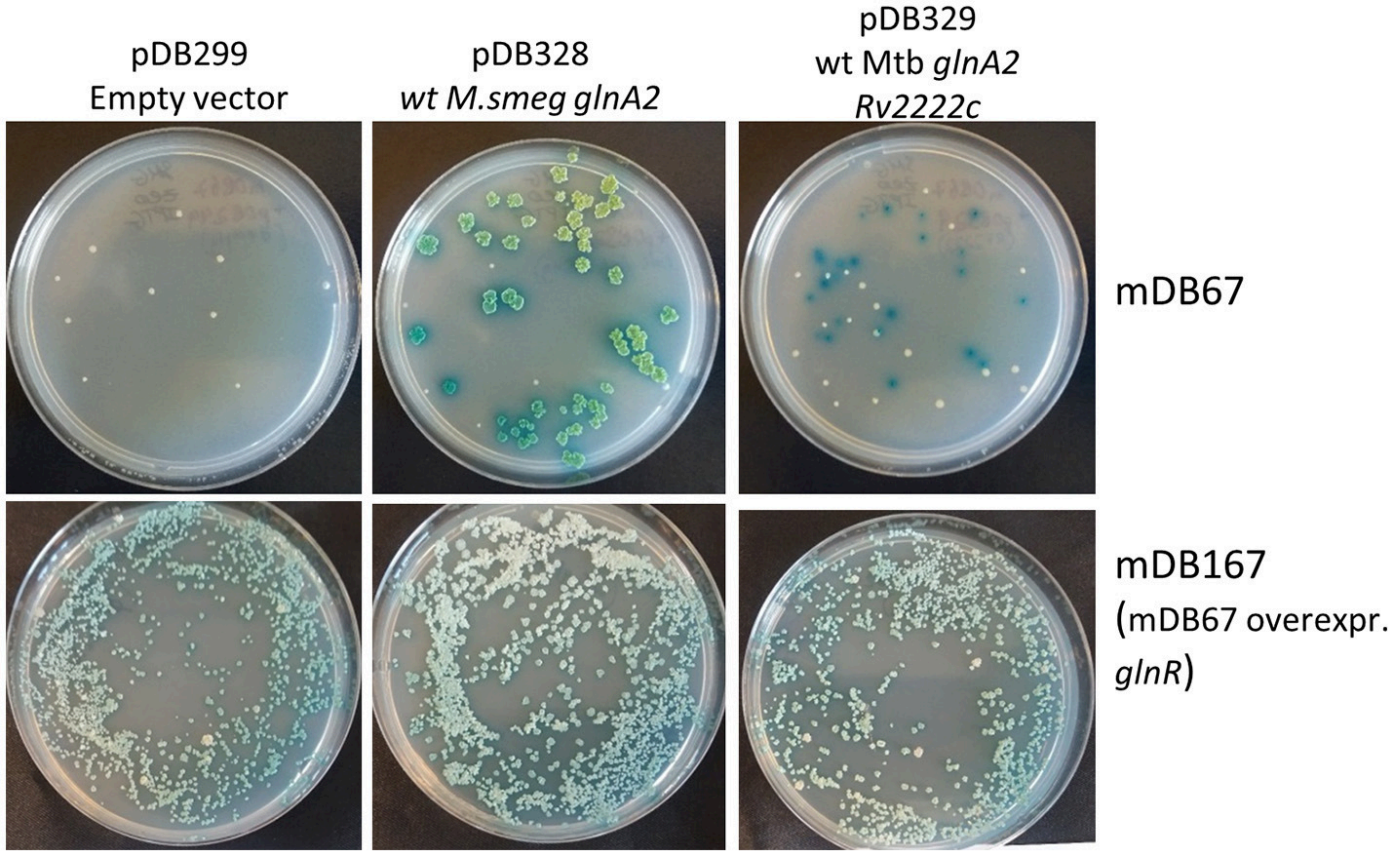
Over-expression of *glnR* allowed for the complete deletion of *glnA2* using cassette-exchange. **Top row:** mDB67 was electroporated by an empty vector, wt *glnA2* or *Rv2222c*. Correct cassette exchanges yielded blue colonies, whereas background colonies are white. **Bottom row:** the same cassette exchanges were attempted in mDB167. Multiple blue colonies arised on the empty vector plate, yielding complete deletion mutants.

To test if this indeed was the factor enabling *glnA2* deletion, we cloned *glnR* with the mutated promoter from mDB76 into a multi-copy episomal plasmid (hygromycin-selected), creating plasmid pDB342. We electroporated pDB342 into mDB67 (*M.smeg*^*I133F*^), creating mDB167. This mutant overexpresses *glnR* due to the mutated promoter and multiple copies of the gene. We then repeated the cassette exchange experiments attempted previously with mDB67, with the newly constructed mDB167. To make true cassette replacement more easily identifiable, we re-cloned the genes into another zeocin-selected empty vector: pDB299, which has *lacZ*, therefore producing blue colonies (when plated on IPTG/Xgal). The three plasmids used were pDB328 (with wt *M_smeg glnA2*), pDB329 (wt *Mtb glnA2, Rv2222c*), or pDB299 (vector control, no complementation). Whereas with mDB67 only pDB238 and pDB329 yielded blue colonies, with mDB167 all three plasmids yielded multiple blue colonies, including pDB299 (empty vector) (Figure 5). The cassette exchange was confirmed by PCR, showing a full deletion mutant of *glnA2* could now be readily created.

**Figure 6:**
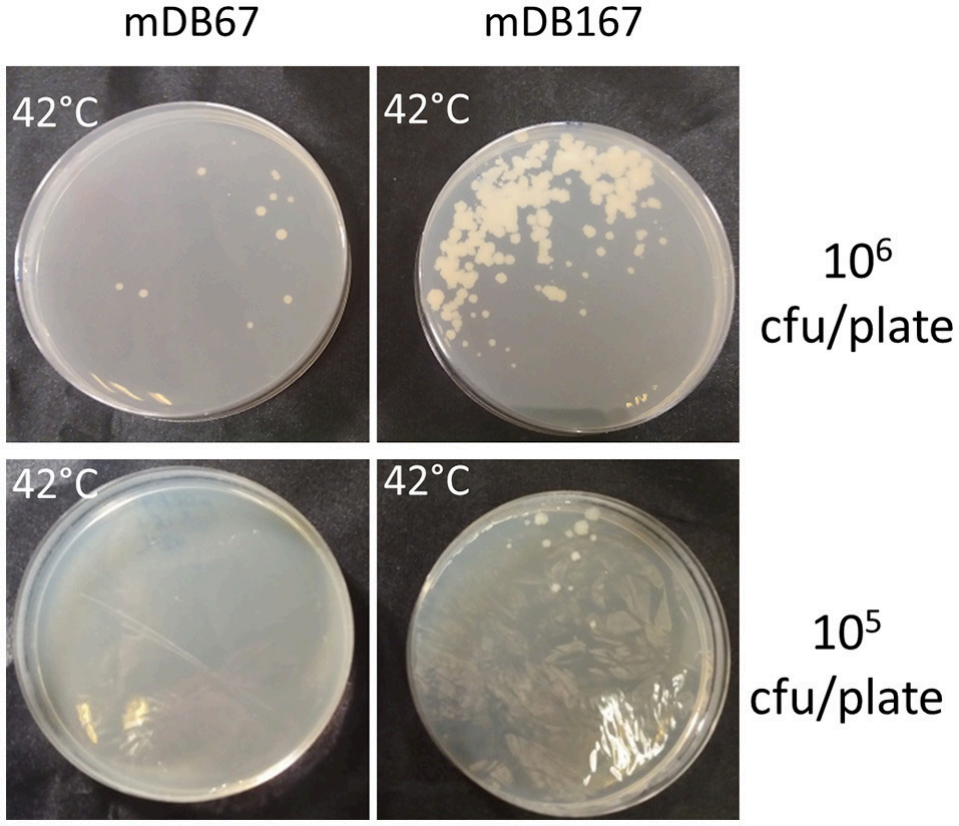
The effect of *glnR* overexpression on “escape” from *glnA2* essentiality. mDB67 and mDB167 were plated in even numbers (10^6^ and 10^5^ cfu/plate) at the non-permissive temperature of 42°C. mDB167 had approximately 10 times more spontaneous “escape” mutants than mDB67.

Since *glnR* is a nitrogen-metabolism regulator, we postulated that its' overexpression does not *per-se* allow the deletion of *glnA2*, but rather that it lowers the threshold for a metabolic adaptation, allowing this deletion. To test this, we plated 10^6^ or 10^5^ cfu/mL of either mDB67 or mDB167, and kept them at 32°C (permissive temperature) or 42°C (non-permissive). If overexpression of *glnR* (in mDB167) would be enough to counter the loss of glnA2, we would expect multiple colonies of mDB167 to arise at 42°C. Instead, most of the growth at 42°C was still inhibited, but we obtained “breakthrough” colonies for both mDB67 and mDB167. However, the number of breakthrough colonies for mDB167 was at least 10-times higher than that for mDB67 (Figure 6). We therefore concluded that a metabolic adaptation compensating for a complete *glnA2* loss (perhaps compendations for lack of D- or iso-glutamine?) was facilitated by high-level *glnR* expression. This implied that another compensatory mutation in nitrogen metabolism pathways may also enable *glnA2* deletion, making a “low threshold” for such mutations.

## Discussion

This study shows that for *M. smegmatis*, unlike in *Mtb, glnA2* is essential, and suggests that it may have an additional role aside iso-, D- or L-glutamine synthesis. The function of *glnA2* is salvaged by high-level expression of *glnR*, a global nitrogen metabolism regulator.

Nitrogen metabolism plays a central role in all bacteria, and its different components are potential drug targets in pathogenic bacteria, including mycobacteria. Glutamine is one of the core substances in nitrogen metabolism, and glutamine synthetases (GSs), are therefore important genes in all bacteria. *M. tuberculosis* has four glutamine synthetases (*glnA1-4*), and several other genes involved in glutamine synthesis and glutamine synthesis regulation (such as *glnE*), but surprisingly, of these four, only *glnA1* (*Rv2220c*) was shown to be essential, and its deletion rendered the bacteria a glutamine auxotroph (Harth et al., 2005; Lee et al., 2006). Mutants deleted in *glnA2, 3*, or *4* were unaffected, were not auxotrophs, and produced *in-vivo* disease in mice indistinguishable from that of the wt (Lee et al., 2006). Although one would expect this to be the situation in other mycobacteria as well, surprisingly, in the very closely related organism *M. bovis*, a *glnA1* deletion was viable even without the addition of glutamine (Chandra et al., 2010), although it did fail to proliferate *in vivo*. However, in that publication other additives were used, with no clear mention of what they were (Chandra et al., 2010). Still, it appears that some of these genes have slightly different roles even in closely related mycobacteria.

In this work, we discovered a spontaneous mutant in *M. smegmatis*, with a phenotype of resistance to an antibacterial chemical with a poorly defined mechanism (O'Donnell et al., 2009), and the mutant had temperature sensitivity. We anticiptaed that identifying the mutation in this strain would shed light on the mechanism of the antimicrobial chemical, and identified the mutation as an I133F mutation in the *glnA2* gene. However, introducing the mutation into the wt did not re-create the resistance phenotype, but did maintain the temperature sensitivity. We therefore took the opportunity to examine the role of *glnA2* in *M. smegmatis*. In *M. tuberculosis* this non-essential gene is thought to be involved in D-glutamine, rather than L-glutamine biosynthesis. However, our experiments convincingly showed that in *M. smegmatis* the *glnA2* gene is essential as its complete removal was not possible, and when grown at a non-permissive temperature, the mutant failed to “keep up” with wt *M. smegmatis* for more than 3-4 generations. More surprisingly, supplementation with neither iso-, L-, nor D-glutamine could salvage the phenotype at the non-permissive temperature, suggesting another role, and not glutamine synthesis, for *glnA2* in these bacteria. However, the *Mtb_glnA2* (*Rv2222c*) could compensate for the loss of *glnA2* in *M. smegmatis*, albeit with a growth defect. The question of which role of *glnA2* in *M. smegmatis* is the essential one, remains to be resolved.

Of note, another possibility is that *glnA2* is indeed responsible for D- or Iso-glutamine synthesis, and that this is the basis for essentiality—but an inability of *M. smegmatis* to use exogenous D- or iso-glutamine is causing the failure of salvage with these AA. L-glutamine auxotrophs of Mtb could be salvaged by exogenous L-glutamine, but it is possible that D- and iso- amino acids behave differently.

*M.smeg_5784*, also known as *glnR*, is an ompR-like regulator-activator of nitrogen metabolism (Jenkins et al., 2013), that was previously shown to control dozens of genes in *M. smegmatis* (and in other bacteria) (Williams et al., 2015; Xu et al., 2017). We have shown here that up-regulation of *glnR* expression can facilitate the emergence of “*glnA2*-deletion” resistance, as the rate of spontaneous mutant-appearance to *glnA2* inactivation (by non-permissive temperature) was 10 times higher in bacteria overexpressing *glnR* compared to that of wt levels. Furthermore, overexpression of *glnR* allowed for the relatively easy creation of a complete *glnA2* deletion-mutant, suggesting that the transcriptomic (resulting in proteomic, and finally –metabolomic) alteration induced by this overexpression favored the metabolic adaptation needed to sustain such a deletion. This is somewhat similar to the situation described in recently published research, where the murL gene was shown to be essential in *M. smegmatis*, and was a D-glutamate auxotroph. However, a point mutation in the promoter of another gene, *Msmeg_5795*, which up-regulated the latter's expression, salvaged the auxotroph phenotype (Mortuza et al., 2018). How exactly the upregulation of *glnR* salvages the essentiality of *glnA2* is not completely clear—this could be by upregulation of metabolic pathways bypassing the bottleneck created by *glnA2* deletion, by inducing a gene with a redundant function or other possibilities. It is also possible that *glnA2* is needed for the proper function of *glnR* itself (by stabilizing it as a dimer), and higher expression of *glnR* compensates for lesser stability of the dimer. A somewhat similar mechanism is present in *Bacillus subtilis* (Fisher and Wray, 2008, 2009; Wray and Fisher, 2008). Also, work in *Paenibacillus riograndensis* showed that the binding of *glnR* to its targets is facilitated when *glnR* is complexed with a glutamine synthetase—again, upregulation of *glnR* may bypass the lack of *glnA2* [Hauf et al., 2016; Fernandes et al., 2017].

It remains unresolved as to what is the reason for *glnA2* essentiality. The possibilities are numerous but could include the depletion of some metabolite, the accumulation of a toxic metabolite due to a metabolic bottleneck, or an essential role in a process such as cell wall remodeling, needed for cell proliferation. Of special interest is the finding that despite great similarity between the GS genes of *Mtb* and *M. smegmatis*, and specifically the *glnA2* gene (which has 83% homology of DNA sequence, and 88% homology of amino acid sequence), found to be non-essential in one is highly important for the physiology of the other. If *glnA2* in *M. smegmatis* does indeed have additional roles over those of *glnA2* in Mtb, it remains to be explored if these roles are unique to *M. smegmatis*, and if not, what are the genes responsible for these functions in Mtb.

### Experimental procedures

#### Bacteria and growth conditions

The *M. smegmatis* used was the standard laboratory-strain mc^2^-155, grown as previously widely described in 7H9 liquid broth or on solid 7H10 plates, both supplemented with glycerol and OADC (and Tween 80 for 7H9 broth). When appropriate, antibiotics were added in the following concentrations: kanamycin 20 μg/mL, zeocin 33 μg/mL, and hygromycin 50 μg/mL. When appropriate, L-glutamine, D-glutamine, or asparagine were added to the growth media at 20 mM (Harth et al., 2005).

#### Whole genome sequencing (WGS)

Genomic DNA was extracted from mycobacteria as previously described, and sent for WGS at the DNA Services Facility in the University of Chicago, Illinois.

#### Construction of an unmarked *glnA2* deletion mutant

To completely delete *glnA2* from mc^2^-155, we first created a merodiploid strain, as described in the results section. We then cloned the 600 bp upstream and downstream flanking regions immediately next to *glnA2* (including the first and last few nucleotides) into the plasmid pAJF013, that also carried the zeocin resistance gene, and the two negative selection markers *galK* and *sacB*. This plasmid was then used to create a complete, unmarked deletion mutant in a two-step allelic exchange technique, as described before (Barkan et al., 2011), and the mutant was named mDB67. The complete deletion of *glnA2* was demonstrated by amplifying the *glnA2* region with primers external to the 600 bp flanking regions. Whereas in wt the PCR reaction yielded a 2,600 bp product, in a full deletion mutant the product was only 1,300 bp long. The 1,300 bp product was gel-purified, sequenced, and shown to contain the upstream and downstream regions of *glnA2*, but not the gene itself. The deletion was later also confirmed by whole genome sequencing.

#### Testing the wt or mutated *glnR*-promoter activity by RT-PCR

We used *sacB* as a reporter gene, and cloned it either under the wt or the mutated *glnR* promoter, using a PCR primer extension, into an *attp*-integrating vector (pDB213). This vector was electroporated into wt *M. smegmatis*, creating the mutants mDB153 (with *sacB* under wt *glnR* promoter) and mDB154 (*sacB* under the mutated promoter). Bacteria were grown to log phase, and RNA was isolated as follows: 1 mL of OD_600_ = 0.4 bacteria were harvested by centrifugation at 3000 g for 1 min and re-suspended in 1 mL of Trizol Reagent (Thermo Fisher Scientific, #15596026). After incubation of 1 min on ice, bust bead-beating was carried out (Precellys Lysing Kit) with 30 s cycles of beating/resting for 10 cycles. The lysate was centrifuged and the RNA extraction was then performed (Pure Link RNA mini Kit # 12183018A). For real-time reverse transcription PCR (qRT-PCR) analysis, one microgram of each RNA sample was reverse transcribed with the High-Capacity cDNA Reverse Transcription Kit (Thermo Fisher # 4368814). Real-time PCR was performed in a StepOne real-time PCR instrument (Applied Biosystems) with SYBR Green PCR Supermix (Invitrogen). The primers for *sacB* were: reverse 5′-GCTGGCCATTACAAAACGCT-3′, forward 5′-GACGATGTGGTAGCCGTGAT-3′; for *glnR*: F: 5′ - GAAGAAGTCGTAACCCC - 3′, R: 5′ - AGTGATCAACGAAGGCG−3′. Normalizing primers: s16-f: 5′-GTGCATGTCAAACCCAGGTAAGG-3′, s16-r: 5′-GGGATCCGTGCCGTAGCTAAC-3′. The results shown are the average of five independent experiments.

A detailed list of plasmids and strains used in this study can be found at Supplementary Material.

## Author contributions

SG, OZ, and DB conceived the design of the study. NR, KG, MB, and DB performed the acquisition, analysis, and the interpretation of the data. NR, SG, OZ, and DB wrote the manuscript.

### Conflict of interest statement

The authors declare that the research was conducted in the absence of any commercial or financial relationships that could be construed as a potential conflict of interest.
