# Supplementary material for: The Unexpected Essentiality of glnA2 in Mycobacterium smegmatis Is Salvaged by Overexpression of the Global Nitrogen Regulator glnR, but Not by L-, D- or Iso-Glutamine
Source: Front Microbiol. 2018 Sep 11;9:2143. doi: 10.3389/fmicb.2018.02143 (PMC6142876; doi:10.3389/fmicb.2018.02143)
Supplement: Supplementary file 1 [file Table_1.DOCX]

**The unexpected essentiality of *glnA2* in *Mycobacterium smegmatis* is salvaged by overexpression of the global nitrogen regulator *glnR*, but not by L- or D-glutamine.**

Rakovitsky N,…. Barkan D.

**Plasmids used in this study**:

| Name | Genes | Selection | Origin | Use |
| --- | --- | --- | --- | --- |
| pDB221 | *glnA2*^mut^ | kanamycin | Attp: integrating | Creating merodiploid mutant for *glnA2*. |
| pDB240 | *glnA2* flanking regions, *sacB, galK* | zeocin | No mycobacterial origin | Deletion of *glnA2* |
| pBRLUX13 | *LUX A-E* | kanamycin | Attp: integrating | Creating a luminescent mutant |
| pDB234 | Empty vector | Zeocin and kanamycin | Attp: integrating |  |
| pDB247 | pDB234+*wt_glnA2* |  |  |  |
| pDB259 | pDB234+*Rv2222c* |  |  |  |
| pDB294 | pDB234+*Rv1878* |  |  |  |
| pDB295 | pDB234+*Rv2220c* |  |  |  |
| pDB299 | *lacZ* | zeocin | Attp: integrating |  |
| pDB328 | pDB299+ *wt_glnA2* |  |  |  |
| pDB329 | pDB299+*Rv2222c* |  |  |  |
| pDB316 | pDB234+*sacB* under *wt glnR* promoter |  |  |  |
| pDB317 | pDB234+*sacB* under mutant *glnR* promoter |  |  |  |
| pDB342 | *glnR* with a mutated promoter | hygromycin | *oriM* (episomal) | To examine the effect of *glnR* overexpression |

**Strains and mutants used in this study**:

| Name of strain/mutant | Genotype | Description/phenotype |
| --- | --- | --- |
| *M. smegmatis* wt (mc^2^-155) |  | Reference laboratory strain |
| mDB67 | *M. smeg* *ΔglnA2; attp:glnA2^I133Fmut^*: kanamycin | Temperature sensitive |
| mDB76 | mDB67+pDB234.  *M. smeg* *ΔglnA2*;attp:zeocin  Spontaneous *glnR* promoter mutation | Full *glnA2* deletion mutant, with mutation in *glnR* promoter. Not temperature sensitive. |
| mDB153 | Wt + pDB316 | Used to compare *glnR* promoter activity, wt versus mutated, by RT-PCR. |
| mDB154 | Wt + pDB317 |  |
| mDB167 | mDB67 + pDB342 | Similar to mDB67, but overexpressing *glnR* |
| mDB149 | Wt + pBRLUX13 | luminescent wt  *M. smegmatis* |
